# Diversity and evolution of the repetitive genomic content in *Cannabis sativa*

**DOI:** 10.1186/s12864-018-4494-3

**Published:** 2018-02-21

**Authors:** Rahul Pisupati, Daniela Vergara, Nolan C. Kane

**Affiliations:** 1 Department of Biotechnology, Indian Institute of Technology, Kharagpur, 721302 India; 2 Ecology and Evolutionary Biology, University of Colorado, Boulder, 80302 USA; 3 Present address: Gregor Mendel Institute, Dr. Bohr-gasse 3, Vienna, 1030 Austria

**Keywords:** Transposable elements, Next generation sequencing, Graph based clustering, Repeat explorer

## Abstract

**Background:**

The repetitive content of the genome, once considered to be “junk DNA”, is in fact an essential component of genomic architecture and evolution. In this study, we used the genomes of three varieties of *Cannabis sativa*, three varieties of *Humulus lupulus* and one genotype of *Morus notabilis* to explore their repetitive content using a graph-based clustering method, designed to explore and compare repeat content in genomes that have not been fully assembled.

**Results:**

The repetitive content in the *C. sativa* genome is mainly composed of the retrotransposons LTR/*Copia* and LTR/*Gypsy* (14% and 14.8%, respectively), ribosomal DNA (2%), and low-complexity sequences (29%). We observed a recent copy number expansion in some transposable element families. Simple repeats and low complexity regions of the genome show higher intra and inter species variation.

**Conclusions:**

As with other sequenced genomes, the repetitive content of *C. sativa*’s genome exhibits a wide range of evolutionary patterns. Some repeat types have patterns of diversity consistent with expansions followed by losses in copy number, while others may have expanded more slowly and reached a steady state. Still, other repetitive sequences, particularly ribosomal DNA (rDNA), show signs of concerted evolution playing a major role in homogenizing sequence variation.

**Electronic supplementary material:**

The online version of this article (10.1186/s12864-018-4494-3) contains supplementary material, which is available to authorized users.

## Background

Repetitive sequences occupy the majority of any typical eukaryotic genome, yet are poorly understood in many respects. The effect of this repetitive content has been under debate for decades [1]. Numerous scientists regard it as parasitic or ‘selfish DNA’ [2]. Others think repetitive elements might play important roles in the host’s genome by altering a gene’s function [3], or by acting as raw material for new genes [4]. These ideas are not mutually exclusive, with repetitive sequences likely having both positive and negative effects in most genomes. Repetitive DNA elements can be mainly classified into two major groups based on their organization in the genome [5]. One group includes sequences showing a tandem repeat organization, where copies are arranged adjacently to each other, commonly (though not always) in or near centromeric and telomeric regions [5, 6]. DNA elements which form tandem arrays such as satellite DNA, simple repeats and ribosomal DNA (rDNA) occur primarily in tandem repeat blocks [5, 7]. A second group of repetitive DNA sequences consist of elements which are dispersed across the whole genome [8]. These include mobile elements like DNA transposable elements (TEs) and retrotransposons such as long terminal repeat elements (LTRs), short interspersed nuclear elements (SINEs), and other dispersed repeats [9, 10].

Eukaryotic species show huge variation in genome size, ranging from only 2.3 Mb in the microsporidian *Encephalitozoon intestinalis* [11] to over 152 Gb in the plant *Paris japonica* [12], five orders of magnitude of variation largely due to changes in repetitive content of the genome, although changes in gene content and ploidy also play a role [13]. Repetitive elements occupy a substantial fraction in most plant genomes, ranging from 20% in *Arabidopsis thaliana* to more than 80% in *Helianthus annuus* (sunflower) [14, 15]. Copy numbers of mobile elements range from thousands to millions per diploid genome [16]. Indeed, just within flowering plants, genome sizes differ roughly by 2500-fold largely due to variation in the copy numbers of TEs and other highly repetitive sequences [17, 18].

The most common repeat sequences fall into several major categories. Due to their copy-paste transposition mechanism, active retrotransposons have the potential to increase their copy number affecting the genome size. LTR/*Gypsy* and LTR/*Copia* are two super-families of retrotransposons present in high copy numbers, resulting in major fractions in flowering plant’s genomes [10]. Usually a smaller proportion of the genome, but highly important, ribosomal DNA (rDNA) encoding sequences in higher plants are arranged in long tandem arrays [7]. Plants typically have 500 to 40000 rDNA copies per diploid cell [19]. Simple sequence repeats, which exhibit high mutation rates, are also abundant within both animal and plant genomes [20]. Together, these classes of sequences make up the majority of the high-copy sequences in most well-characterized eukaryotic genomes.

Some classes of repetitive elements, such as LTR elements, provide an opportunity for deciphering the evolutionary demography of a family of retrotransposons within host genomes. Newly produced retrotransposons are 100% identical to the parental molecule but with no mechanism to maintain their homogeneity after insertion, they are expected to diverge neutrally [18]. Mutations gradually disfigure the elements to different lengths leading to an incomplete structure that might also inactivate them [21]. The magnitude of pairwise divergence between two LTRs can be used to infer their relative ages [22]. Studies on LTR families in rice [23], maize [22] and peas [24] calculated insertion ages using pairwise divergence among the elements and found that the average level of divergence for a large fraction of LTR elements is on the order of 1% or less [18]. These genomes thus are largely composed of recently duplicated repeat sequences.

*Cannabis sativa* (marijuana, hemp), a member of the family Cannabaceae is a widely cultivated plant with numerous genomic questions unanswered. The family Cannabaceae has 11 genera with approximately 100 species widely distributed throughout Asia and Europe [25]. *Cannabis sativa* is one of the earliest domesticated and cultivated plant species for fibre, oil, and for its medicinal and psychoactive properties [26]. *Cannabis sativa* has a diverse set of metabolic compounds as cannabinoids and terpenoids [27] and the numerous varieties vary morphologically in the production of these compounds [28]. *Cannabis sativa* Purple Kush (PK) is a commonly used recreational marijuana variety, while the cultivars ‘Finola’ and ‘USO31’ are hemp varieties used industrially [29]. These three varieties were sequenced in 2011 [30] and their sequences are publicly available at NCBI’s short read archive. The estimated size of the haploid *C. sativa* genome is 830 Mb and the draft genome assembly contains approximately 80% of the estimated genome [30]. In order to understand more about the genome of this important species, we studied its genomic diversity by exploring the TE dynamics.

*Humulus lupulus* (hops), the closest relative of *C. sativa*, has a genome three times as big (2570 Mb) [31]. *Morus notabilis* (mulberry), a closely related species to both *C. sativa* and *H. lupulus* diverged approximately 63.5 MYA [32] and was the closest related species of *C. sativa* sequenced until recently. The estimated genome size of *M. notabilis* is 357 Mb, which is less than half of *C. sativa*’s genome [32]. Thus, these genera span a relatively large range of genome size variation. Using the genomes of these three related genera allows us to make comparisons between them to explain the variation in genome size within and among species of Cannabaceae family.

## Results

### Characterizing repetitive content in genomes

**Fig. 1:**
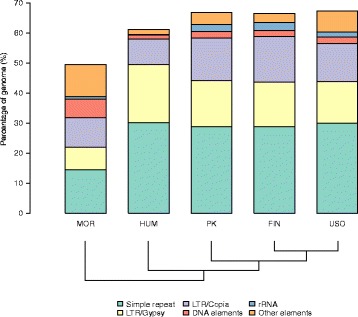
Genome characterization by Repeat explorer. The graph based clustering algorithm (RE) characterized 64.5%, 64.5%, 65.2%, 60.1% and 43.3% of genome to be most high and medium copy number regions in *M. notabilis* (MOR), *H. lupulus* (HUM), *C. sativa* PK, Finola (FIN), and USO31 (USO), respectively. Cladogram modified from van Bakel et al. [30]

We determined that the repetitive content, characterized using Repeat Explorer (RE) [33]. Repeats occupy 64-65% of each *C. sativa* genome, 43% of the *M. notabilis* genome and 60.1% of the *H. lupulus* genome (Fig. 1). The analysis revealed LTR/*Gypsy* and LTR/*Copia* retrotransposons to be particularly abundant (ranging from 12-15%, 7-10% and 8-19% in *C. sativa*, *M. notabilis*, and *H. lupulus* respectively). LTR/*Copia* retrotransposons are mainly represented by *Angela* and *AleII* lineages, while LTR/*Gypsy* by *Ogre/Tat*, *Athila* and *Chromovirus* lineages. Simple and low complexity repeats were also found in high content (approximately 27%, 14.3% and 29% in genomes of *C. sativa*, *M. notabilis* and *H. lupulus* respectively). rDNA occupies approximately 1.7-2.5%, 0.9% and 0.1% in *C. sativa*, *M. notabilis*, and *H. lupulus* genomes respectively (Additional file 1: Table S2). As a validation, we analyzed a well annotated genome of *Arabidopsis thaliana* using RE, which is consistent with our results (Additional file 1: Table S1). Also we included the genome repeat characterization of lineages in other Cannabis varieties, which are similar to those of PK, USO31, and Finola (Additional file 1: Table S1).

**Fig. 2:**
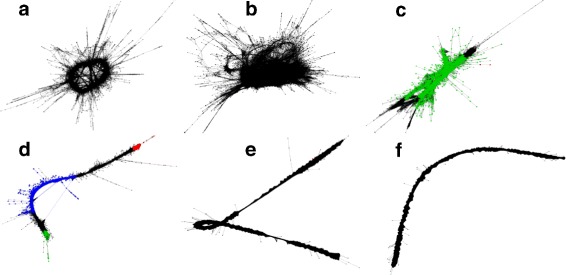
Graph layouts. Clusters of simple repeats (**a** and **b**), LTR/*Gypsy* (**c**), LTR/*Copia* (**d**) rDNA (**e** and **f**), and in *C. sativa*’s PK genome. Colors indicate different domains (*Ty1-INT*, *Ty1-RT* and *Ty3-RT* in red, blue and green respectively) of LTR elements

RE clusters sequences based on their pairwise similarity and generates graphs for each cluster using all-to-all pairwise comparisons. Each graph is similar to a de Brujjn graph [34] where every vertex correspond to a sequence, and their pairwise similarity score is expressed as edge weight [35]. The graph topology of the cluster can give information about the type of the repetitive element. In Fig. 2a and b, graphs are less dense and have larger diameter which contains simple repeats and low complexity. Graphs with long units like LTR retrotransposons are characterized by the presence of multiple LTR domain hits (Fig. 2c and d), which produce linear structures when the nodes connect densely between them into threadlike structures [35]. Full length LTR elements also produce circular layouts similar to the ones in Fig. 2c and they have annotated LTR domains.

Usually rDNA graphs exhibit a circular layout due to their tandem organisation [35]. However, our results (Fig. 2e and f) displayed densely connected linear arrangement which are due to the lack of sequencing coverage [35] at the ends of the rDNA repeats. This is likely due to variation in sequence among repeats as observed by Novak et al. 2010 [35]. If that is the case, it suggests that the repeats are evolving concertedly across most of their length, but that repeats vary in the sequence of the spacer regions.

### Divergence calculations

**Fig. 3:**
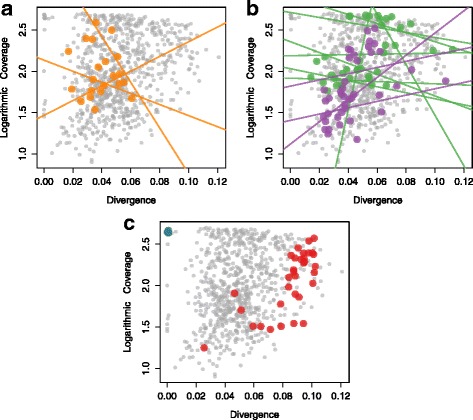
Transposable elements in the PK genome and their amplification. Linear regresssions between the pairwise divergence of the TEs within the PK genome (x-axis), and each TE family’s copy number (y-axis). Sequences clustered into the **a** LTR/*Copia* (orange), **b** LTR/*Gypsy* (*Chromovirus* and *Athila* in green and purple respectively), **c** rDNA (blue) and simple repeats (red) respectively. The remaining TEs in clusters are shown in grey dots

We performed a *de novo* assembly of sequencing reads in clusters using RE that produced varying numbers of repeat family sequences in the different genotypes. We selected the sequences with a minimum length of 500 base pairs and subsequently filtered the library to remove plastid and virus sequences, which resulted in a repeat library (a set of sequences generated from highly repetitive genomic sequences) of 803 contigs in *C. sativa* PK, 548 in USO31, 854 in Finola, 424 contigs in *H. lupulus* and 396 contigs in *M. notabilis*. Coverages across the contigs, which measure the number of copies of a particular element present in the genome, range from as high of 500 to as low as 8 copies per genome in some repeats (Fig. 3). In order to understand whether the elements with more copies were more divergent, we plotted the average pairwise divergence between those contigs and their respective coverages (Fig. 3).

The pairwise divergence measure, *θ*_*π*_, is widely distributed in the repetitive sequences of *C. sativa*’s genome varying from 0.11 to as low as 0 in terms of sequence divergence. The rDNA sequences present in the genome have very low pairwise divergence (average of 0.00327) with high coverages, occupying the top left portion in Fig. 3. This suggests rDNA sequences are conserved within the three varieties of *C. sativa*. On the other hand, simple repeats and low complexity found on the right portion in the plot have high pairwise divergences, which suggest that these sequences accumulate mutations at a higher rate.

**Fig. 4:**
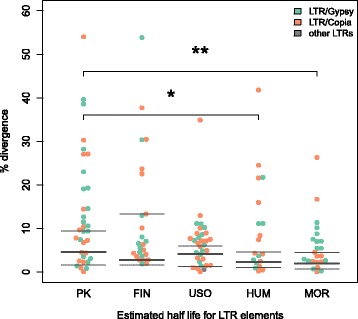
LTR element divergence in the genomes. Median, 25th, and 75th percentiles of the estimated half-life for elements in clusters for *C. sativa* PK, Finola (FIN), USO31 (USO), *H. lupulus* (HUM) and *M. notabilis* (MOR). Asterisks mark significant difference (Wilcox - Mann - Whitney Test, * indicate *p*-value ≈ 0.1 and ** indicate *p*-value <0.05)

We calculated the estimated half-life for LTR elements present in clusters for *C. sativa* PK (PK), Finola (FIN), USO31 (USO), *M. notabilis* (MOR) and *H. lupulus* (HUM) (Fig. 4) as detailed in the methods section. We determined the median and standard deviation of the estimated half-life for LTR clusters in all the genomes. PK has the highest median (0.1002; s.d. = 0.331) of our analyzed genomes, followed by USO31 (0.0667; s.d. = 0.537), Finola (0.0643; s.d. = 0.351), *H. lupulus* (0.0428; s.d. = 0.11), and *M. notabilis* (0.0408; s.d. = 0.14) in percentage sequence divergence. We also performed an ANOVA on the R statistical framework. The differences in the half-life among the genomes is non-significant (F = 0.596, *p* = 0.67). We used a Wilcox-Mann Whitney rank test to understand whether differences for each pair of genomes were significant. We found that PK differs from MOR (0.049, *p* <0.05) but the difference is not significant compared to HUM (0.035, *p* = 0.1266), USO (0.038, *p* = 0.0222) and FIN (0.015, *p* = 0.5376). *Morus notabilis* also showed significant differences from FIN (-0.025, *p* = 0.0633) but not to USO (-0.009, *p* = 0.4082) or to HUM (-0.007, *p* = 0.6026).

### Network representations

**Fig. 5:**
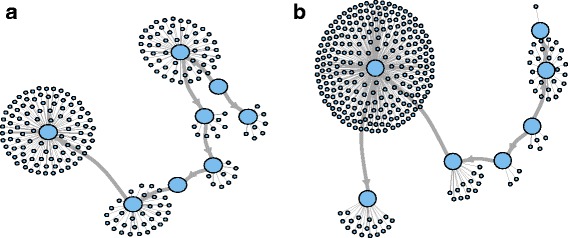
Network representations using AnTE. LTR elements present in *C. sativa* PK genome represented in a phylogenetic network. All sequences are represented as nodes and their ancestor-descendant relationships are indicated by the arrows. **a** LTR/*Gypsy*. **b** LTR/*Copia*

We described a detailed picture of the TE evolutionary history using the method AnTE [36], which also provides ancestral sequences. We show network representations of the relationships among the elements of LTR/*Gypsy*, LTR/*Copia* and for rDNA elements (Fig. 5). The LTR/*Gypsy* elements present in one of the clusters of the *C. sativa* PK genome, are shown in Fig. 5a. For the LTR/*Copia* elements, most of the sequences (Fig. 5b) have a single ancestral sequence from which populations diverged. This ancestral sequence is represented as a large circle from which arrows leave to form new sequences, suggesting a recent population expansions. In other words, after diverging from a single insertion event, each element proliferates to generate multiple copies of the same sequence in the genome.

### Comparative analysis among species

Clustering analysis on a combined dataset of the five genomes shows that 11.54%, 9.81%, 9.41%, 6.42% of Finola, USO31, *M. notabilis* and *H. lupulus* genomes have equivalent clusters in the *C. sativa* PK genome respectively. We calculated the similarities in the repetitive content and across the TE families with respect to *C. sativa* PK genome.

It has been previously shown that *M. notabilis* and *C. sativa* diverged about 63.5 MYA, while *Cannabis* and *Humulus* diverged more recently [32]. Consistently, the repetitive sequence library in *M. notabilis* is the most divergent (Additional file 1: Table S3). The rDNA sequences are more conserved and their homologous copies in the genomes of *M. notabilis*, USO31, and Finola have divergences of 32%, 19%, and 3% respectively. The paralogous copies of rDNA in the PK genome have an average divergence rate of 0.3%, showing signs of concerted evolution. We also found that sequences for simple and low complexity repeats have considerable variation in percent similarity among the three species.

## Discussion

Genome size variation can be partially explained by its repetitive content. We found that 64% of *C. sativa* genome is repetitive, which is less when compared to Maize (85% [37] and Sunflowers (80% [38]) that have larger genomes, 2.3 Gb and 3.5 Gb respectively. However, despite substantial differences in genome size, proportions of repetitive content in species related to *C. sativa* are comparable, including *H. lupulus* (60.1%), *M. notabilis* (47% [32]), and *M. domestica* (67% [39]). Thus, some differences in genome size are associated with quantifiable repeat content differences, but it is not the only component explaining genome size. Polyploidy, and smaller scale duplications also play a role [13] and these cannot be detected by RE. The repetitive content in *H. lupulus* is approximately 60.1%, which is much lower compared to Maize and Sunflower that have bigger genomes, but smaller than *C. sativa* despite its the much larger genome size. These results, though surprising, are similar across multiple lineages within the species: 60.1% for *H. lupulus* Japanese wild hops, 61.3% for var. Lupulus, and 59.2% for var. cordifolius. The analysis of *A. thaliana* rules out artifacts from the pipeline (Additional file 1: Table S1). Additional, ongoing work, aims to better understand the repeat content in *Humulus*.

The majority of *C. sativa*’s genome consists of LTR/*Copia*, LTR/*Gypsy* and simple repeats, unsurprisingly since LTR elements are present in high copy numbers in many flowering plant genomes [10]. The pairwise divergences across the elements can be used to calculate the insertion times of each TE family [9, 18, 23]. In Fig. 3, we show the logarithmic coverage (y-axis) against the pairwise divergence (x-axis) across the elements, with the corresponding linear regressions for each cluster. Using the slope of the linear regressions, we estimated the half-life for the LTR elements in terms of sequence divergence. For some of the LTR/*Gypsy* elements, the half-life distributions suggest that 50% of the insertions are lost by the time they have diverged by an average of 8% at the nucleotide sequence level. On the other hand, LTR/*Copia* elements seem to survive much longer in the *C. sativa* genome, with an average half-life divergence of 15%. LTR elements in other plants such as rice [40], maize [9] and wheat [41] seem to have lower half-life estimates, suggesting higher rates of turnover. The relatively high ages of LTR families in *Cannabis* may indicate a stable genomic content, with little recent turnover in repeat content.

In addition to estimating the half-life, the slope of the linear regressions in the divergence plot (Fig. 3) can reveal population dynamics and the type of selection present in the elements. A negative slope for the linear regression suggests that the element is under neutral selection [9, 18], because elements gradually accumulate mutations rendering them inactive. Some clusters that have a positive slope could either be under directional selection since they are not following a steady birth-death model. A positive slope can also signify a recent population expansion, meaning that sequences proliferate after diverging. This recent increase in copy number in a type of element can be confirmed with the phylogenetic relationship calculated through AnTE (Fig. 5b). The network analysis shows that many new insertions arose from an ancestral sequence for some LTR/*Gypsy* elements in the *C. sativa* genome. Since we already know that some of these repeat families are unique to the derived Y chromosome in *Cannabis* [42–44], it will be interesting to investigate the functional significance, if any, of other repeat families, particularly as genetic maps and other resources become better developed in this species.

Relating transposon sequences and copy number to phenotypic traits is often challenging. However, it is known that in *Cannabis*, retroposons are associated with sex differences among males and females [42–44]. The analysis presented here is solely on females, but ongoing work on additional genotypes will further explore repeat content differences among males and females, as well as investigating copy number associations with other important traits, such as the production of secondary metabolites (cannabinoids) many of them with medicinal properties [45, 46].

In most eukaryotes, rDNA genes are present in tandem repeated arrays in high copy numbers [47]. The copy number of rDNA genes varies between species, and in *A. thaliana* approximately 10% of the genome size variation is due to the differences in rDNA gene copies [48]. We found that 2% of the *C. sativa*’s genome is composed of rDNA genes (Additional file 1: Table S2). We also found that *H. lupulus* has approximately 0.1% of its genome occupied by rDNA genes, with fewer copies despite its larger genome size. Unsurprisingly, we found that rDNA elements present in the *C. sativa* genome show signs of concerted evolution, as expected based on substantial work in other species [49–51]. However, a handful of other repeat sequences also showed similar levels of concerted evolution (Fig. 3).

The other major type of repeats found in the non-coding part of the genome are simple sequence repeats, microsatellites or low-complexity regions [52], and they occupy approximately 25% of *C. sativa*’s genome, 13% of *M. notabilis* genome, and 30% of *H. lupulus* genome. Since we estimated the repeat content from raw sequence reads, our estimate is unbiased. Additionally, our estimate of these repetitive elements in *M. notabilis* genome is consistent with previous findings [32]. These sequences show high divergence across species, as expected considering the fact that these regions have higher mutation rates [52].

## Conclusions

Our study gives insight into the composition and the dynamics of repeats in the genomes of three varieties of *C. sativa* and two related genera. Among the three genera, we found similar repetitive content. The prevalence of LTR/*Copia* and LTR/*Gypsy* elements in the three genomes of *C. sativa* and *H. lupulus* is higher than in *M. notabilis*, which may partially explain the variation in the genome sizes. Our estimated half-life for LTR/*Copia* elements is higher compared to LTR/*Gypsy* elements in the *C. sativa* genome, showing higher turnover in these elements. rDNA elements, as well as some other unclassified repeats, show signs of concerted evolution, and some LTR elements show recent population expansion. Finally, the network representations for the repetitive elements present in genome validate the population expansion in the LTR elements, and help to explain the proliferation of these major components in the genomes.

## Methods

### Determining repetitive content

We analyzed the raw genomic reads obtained from the published genome of three different *C. sativa* varieties PK, USO31, and Finola [30], mulberry (*M. notabilis*) [32] and hops (*H. lupulus*) [31], using a graph based clustering pipeline. This method requires high-throughput genome sequencing data and does not require an assembled genome for characterizing the repetitive content. Genome shotgun sequencing reads were taken from NCBI’s Short Read Archive (SRA) [SRR352164], [SRR351494], [SRR351929], [SRR847535] and [DRR024456] for the three *C. sativa* varieties, *M. notabilis* and *H. lupulus* respectively. To confirm the repeat content estimates for *H. lupulus*, which were surprisingly low given the large genome size of the species, we also analyzed Japanese wild hop, var. Lupulus and var. cordifolius within SRA database [DRR024456], [DRR024410], [DRR024452]. We also analyzed four other varieties of *C. sativa* (NCBI accession numbers SRR3294442, SRR3294438, SRR3294431, and SRR3294475 (Additional file 1: Table S1) [53], and a genome of *A. thaliana* [SRR519656] [48] using the same pipeline, to validate the pipeline.

We did trimming and quality filtering of the raw reads using the fastx-toolkit with a quality threshold of 30 and minimum read length of 80. Reduced sets of randomly selected genomic reads for the four genomes (1x coverage) were separately subjected to clustering using RE [33] with default parameters. We used RepBase libraries (accessed 31 January 2014) of *Viridiplantae* and Conserved Domain Database, which contains protein domains derived from plant mobile genetic elements to annotate and classify repeat family of clusters.

### Pairwise divergence between elements

RE is a graph based clustering algorithm, where the sequences are clustered based on their similarity. RE produces contigs by assembling sequences from each cluster that serve as reference sequences and represent TE elements present in genome. We established a library with consensus contigs of each repeat class form each genome, with a minimum sequence length of 500bp. We annotated these contigs using RepeatMasker and Repeat library (accessed 31 January, 2014). We used Burrows-Wheeler Aligner (bwa) [54] to align the genomic reads back to these contigs with default parameters. We calculated the average pairwise divergence for sequences aligned in each contig using Popoolation [55] from a pileup file generated using SAMtools (version 0.1.15) with default parameters [56]. We determined average depth across the contig from the pileup file using custom perl scripts (hosted on GitHub, https://github.com/rbpisupati/ExploringTEsinCannabisGenome/), which is considered to be the copy number of that particular TE family present in the cluster. We calculated the half-life of TEs as measured by sequence divergence (as a proxy for age), assuming a steady-state birth-death model. The half-life is a linear function with the slope of a linear regression line in a log-arithmetic plot between the pairwise divergence and the copy number. We performed a linear regression analysis (lm), a Wilcox - Mann - Whitney analysis, and a t-test in the R statistical framework (version 3.1) with default parameters.

### Phylogenetic networks

Approximately eight million sequence reads in each genome were aligned to the consensus repeat library. Based on the TE annotation of the contigs, we extracted reads aligned to a position in the contig. In order to infer the dynamics and TE ancestry, we used a Bayesian method, AnTE [36] on those aligned sequences with default parameters. AnTE reconstructs the ancestral relationships among the sequences.

### Divergence across the species

To establish the differences between the species, we performed an inter-species comparative analysis of the genomes using RE. We identified homologous sequences between the repeat library of each pair of genomes using NCBI BLAST [57]. We also determined the divergence for TE families across the genomes using the percent similarity from BLAST [57].

## Additional file

Additional file 1 **Table S1**. Raw data information and repeat content for different varieties of *Cannabis sativa*, *Humulus lupulus*, *Morus notabilis* and *Arabidopsis thaliana*. **Table S2**. Repetitive content (percentage of the genome) in the genomes of *C. sativa* PK, Finola (FIN), USO31 (USO), *H. lupulus* (HUM) and, *M. notabilis* (MOR). **Table S3**. Repeat sequence conservation, as measured by percent sequence similarity among consensus sequences from each repeat class in each genotype, compared to the consensus repeats from the *C. sativa* PK genome. The missing entries are due to the absence of specific families in either of the genomes’ repeat libraries. (PDF 163 kb)

## Abbreviations

HUM: *Humulus lupulus*
LTR: Long terminal repeat
MOR: *Morus notabilis*
MYA: Million years ago PK: *Cannabis sativa* purple kush variety
RE: Repeat explorer
rDNA: Ribosomal DNA
TE: Transposable elemen
USO: *Cannabis sativa* USO31
