# Supplementary material for: Diversity and evolution of the repetitive genomic content in Cannabis sativa
Source: BMC Genomics. 2018 Feb 21;19:156. doi: 10.1186/s12864-018-4494-3 (PMC5822635; doi:10.1186/s12864-018-4494-3)
Supplement: Supplementary file 1 — Table S1. Raw data information and repeat content for different varieties of Cannabis sativa, Humulus lupulus, Morus notabilis and Arabidopsis thaliana. Table S2. Repetitive content (percentage of the genome) in the genomes of C. sativa PK, Finola (FIN), USO31 (USO), H. lupulus (HUM) and, M. notabilis (MOR). Table S3. Repeat sequence conservation, as measured by percent sequence similarity among consensus sequences from each repeat class in each genotype, compared to the consensus repeats from the C. sativa PK genome. The missing entries are due to the absence of specific families in either of the genomes’ repeat libraries. (PDF 163 kb) [file 12864_2018_4494_MOESM1_ESM.pdf]

Additional file

Diversity and Evolution of the Repetitive Content in the Genome of *Cannabis sativa*

Rahul Pisupati[1], Daniela Vergara[2], Nolan C. Kane [2§]

Table S1

Raw data information and repeat content for different varieties of *Cannabis sativa*, *Humulus lupulus*, *Morus notabilis* and *Arabidopsis thaliana*.

Species	Strain/Cultivar	Approx. Genome Size (Mbp)	SRARun	Read Length (bp)	Number of reads analysed	MBases analysed	Avg. coverage	Est. Repeat Content
Cannabis sativa	Purple kush	830	SRR352164	100	8000000	800	0.96	64.5
Cannabis sativa	finola	830	SRR351929	100	8000000	800	0.96	64.5
Cannabis sativa	USO31	830	SRR351494	100	8000000	800	0.96	65.2
Cannabis sativa	G13	830	SRR3294442	148	2947748	436	0.53	61.5
Cannabis sativa	Hawaiian	830	SRR3294438	148	4394648	650	0.78	61.3
Cannabis sativa	Carmagnola	830	SRR3294431	148	17595110	2,604	3.14	59.7
Cannabis sativa	Tangerine haze	830	SRR3294475	148	21154734	3,131	3.77	60.1
Humulus lupulus	cordifolius	2500	DRR024456	75	70000000	5,250	2.10	60.1
Humulus lupulus	cordifolius	2500	DRR024452	75	65213792	4,891	1.96	59.2
Humulus lupulus	lupulus SW	2500	DRR024400	109	50000000	5,450	2.18	65.3
Humulus lupulus	lupulus SW	2500	DRR024410	100	50000000	5,000	2.00	61.3
Morus notabilis		330	SRR847535	100	6600000	660	2.00	43.3
Arabidopsis thaliana	Ost-0	150	SRR519656	100	2000000	200	1.33	23.9

Table S2

Repetitive content (percentage of the genome) in the genomes of *C. sativa* PK, Finola (FIN), USO31 (USO), *H. lupulus* (HUM) and, *M. notabilis* (MOR).

	PK	FIN	USO	HUM	MOR
**Total content**	64.50	64.46	65.20	60.10	43.30
**ARTEFACT**	0.17	0.00	0.10	0.00	0.00
**DNA**	0.02	0.00	0.03	0.00	0.00
**DNA/*CMC-EnSpm***	1.39	1.05	1.33	0.98	1.21
**DNA/*hAT***	0.00	0.00	0.00	0.00	0.00
**DNA/*hAT-Ac***	0.02	0.01	0.01	0.03	1.13
**DNA/*hAT-Tag1***	0.00	0.00	0.00	0.00	0.00
**DNA/*hAT-Tip100***	0.00	0.00	0.00	0.00	0.09
**DNA/*MULE-MuDR***	0.81	1.01	0.79	0.37	3.35
**DNA/*PIF-Harbinger***	0.00	0.01	0.00	0.01	0.25
**DNA/*TcMar-Stowaway***	0.00	0.00	0.00	0.00	0.14
**LINE/*L1***	0.15	0.13	0.03	0.11	0.13
**LINE/*RTE-BovB***	0.00	0.00	0.00	0.00	0.18
**Low complexity**	2.87	1.06	3.28	0.89	1.40
**LTR**	0.65	0.44	0.54	0.10	0.00
**LTR/*Caulimovirus***	0.00	0.00	0.00	0.04	0.27
**LTR/*Copia***	14.10	15.14	12.53	8.53	9.80
**LTR/*Gypsy***	15.44	14.85	13.96	19.24	7.57
**RC/*Helitron***	0.02	0.00	0.00	0.00	0.14
**rDNA**	2.25	2.57	1.73	0.10	0.89
**Satellite**	0.08	0.09	0.18	0.12	0.00
**Satellite/centr**	0.00	0.00	0.00	0.00	0.00
**Simple repeat**	25.88	27.75	26.63	29.18	13.04
**Unknown**	0.64	0.36	4.06	0.00	3.72

Table S3

Repeat sequence conservation, as measured by percent sequence similarity among consensus sequences from each repeat class in each genotype, compared to the consensus

repeats from the *C. sativa* PK genome. The missing entries are due to the absence of specific families in either of the genomes' repeat libraries.

	MOR	HUM	FIN	USO
**Average percent similarity**	16.50	24.67	71.71	53.19
**ARTEFACT**	-	-	-	11.38
**DNA/CMC-EnSpm**	14.30	57.21	73.87	53.18
**DNA/hAT-Ac**	-	-	97.07	-
**DNA/MULE-MuDR**	6.52	18.05	73.31	56.41
**LINE**	-		35.73	-
**Low complexity**	0.00	-	19.24	19.75
**LTR**	-	-	19.48	22.81
**LTR/*Copia***	8.75	18.05	76.37	51.14
**LTR/*Gypsy***	13.05	19.04	68.10	49.50
**rDNA**	68.25	-	97.06	81.35
**Simple repeat**	13.62	8.82	75.74	42.64
